# First-principles study on enhancing the photocatalytic hydrogen evolution performance in Cs_3_Bi_2_I_9_/MoS_2_ heterostructure with interfacial defect engineering

**DOI:** 10.1039/d5ra05294g

**Published:** 2025-10-02

**Authors:** Kyong-Mi Kim, Yun-Sim Kim, Dok-Ho Hyon, Chol-Hyok Ri, Chol-Jun Yu

**Affiliations:** a Computational Materials Design, Faculty of Materials Science, Kim Il Sung University Taesong District Pyongyang Democratic People's Republic of Korea cj.yu@ryongnamsan.edu.kp; b Institute of Nano Engineering, State Academy of Science Rakrang District Pyongyang Democratic People's Republic of Korea; c Faculty of Physics, O Jung Hup Chongjin University of Education Chongjin North Hamgyong Province Democratic People's Republic of Korea

## Abstract

Hydrogen has been attracting continuously growing interest as a highly efficient and clean energy source for replacing fossil fuels in the future, and thus developing highly efficient photocatalytic materials for hydrogen evolution is much desirable. In this work, we study the structural, electronic and optical properties of heterostructures composed of the bismuth-based vacancy-ordered iodide double perovskite Cs_3_Bi_2_I_9_ and a two-dimensional dichalcogenide 2H-MoS_2_ monolayer without and with a vacancy defect using first-principles calculations. Our calculations demonstrate that the Cs_3_Bi_2_I_9_/MoS_2_ heterostructures are energetically stable and induce an interfacial dipole moment, which is beneficial for the prevention of charge carrier recombination. Due to the proper band-edge alignment and the smallest Gibbs free energy difference for hydrogen adsorption, the defective interface with a Cs-vacancy (V_Cs_) is found to be the most promising for photocatalytic hydrogen evolution. Moreover, we find that the interfacial V_Cs_ defect can be formed favourably under the I-rich/Cs-poor condition, where V_I_ and V_S_ formations are suppressed. This work provides a way to develop high-performance photocatalysts based on heterostructures composed of the Bi-based halide perovskites and transition metal dichalcogenides for hydrogen evolution from solar-driven water splitting.

## Abstract 2

We studied the interfacial properties and the photocatalytic hydrogen production performance of bismuth-based perovskite Cs_3_Bi_2_I_9_/MoS_2_ heterostructures with vacancy defects using first-principles calculations.

## Graphical abstract

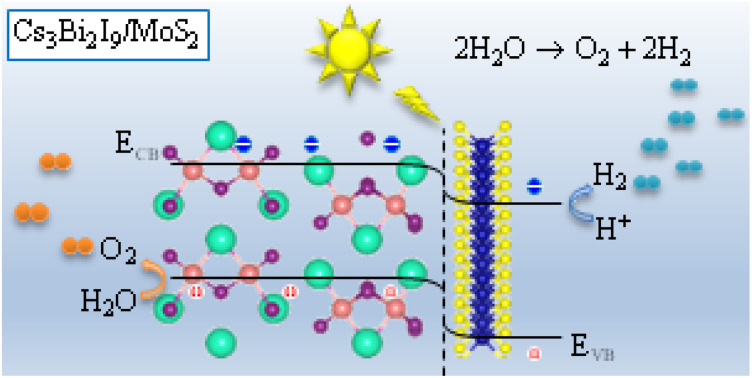

## 1 Introduction

Superseding natural resources with clean sustainable energy sources has become an extremely urgent issue to meet the increasing demand for energy and achieve net-zero carbon emissions in light of the rapid exhaustion of fossil fuels and acute environmental problems. In this context, hydrogen has been attracting continuously growing interest as a highly efficient and clean energy source for the future.^1^ Among various methods for producing hydrogen, solar-driven photocatalytic hydrogen evolution is the most attractive approach in terms of energy balance and clean production due to solar energy utilization.^2–4^ Photocatalysts for hydrogen evolution reactions (HERs) reported so far include carbon nitrides,^5–7^ metal oxides,^8,9^ metal sulfides,^10–12^ organic materials,^13,14^ and heterojunctions.^15,16^ When using those photocatalysts, however, the solar-to-hydrogen (STH) conversion efficiencies are still far below the target threshold of ∼10% required for commercialization.^17^ To increase the STH efficiency towards 10%, many strategies have been developed, including the search for new materials, heteroatom doping, interface engineering and phase engineering.^18^

Since their first use as light absorbers in solar cells in 2009,^19^ halide perovskites (HPs) have been attracting significant attention in the field of photovoltaics, with high power conversion efficiencies exceeding 27% being reported.^20–23^ This is due to the superior optoelectronic properties of HPs, including strong light absorption,^24^ tunable bandgap,^25^ long carrier lifetime,^26^ long carrier diffusion lengths^27,28^ and high carrier mobility.^29^ The best-known photocatalysts for HERs are lead-containing HPs,^30^ which are usually used in the form of heterojunctions with other photocatalysts to prevent the serious recombination of photogenerated charge carriers. As reported by Zhao *et al.*,^31^ the organic–inorganic hybrid HP MAPbI_3_ (MA = CH_3_NH_3_, methylammonium), when integrated with a MoS_2_ monolayer, demonstrated a STH efficiency of 1.09% and prominent hydrogen generation activity (HGA) of 13.6 mmol g^−1^ h^−1^. By integrating a polyfluorene co-catalyst onto the surface of MAPbI_3_, Pal *et al.*^32^ reported a HGA of up to 6.2 mmol g^−1^ h^−1^ in HI solution, almost 200-fold higher than that of pristine MAPbI_3_. In spite of such outstanding photovoltaic and photocatalytic properties, the organic–inorganic hybrid HPs have suffered from poor stability due to the existence of the organic moiety.^33^ Therefore, the organic species (*e.g.*, MA) were replaced with inorganic ones; for instance, CsPb(Br_1−*x*_I_*x*_)_3_ with a noble metal co-catalyst of Pt demonstrated a HGA value of 1.12 mmol g^−1^ h^−1^ in the HI/HBr mixed solution.^34^

In the Pb-based HPs, toxicity of lead is another problem, causing environmental and health care concerns. To address these problems, several metals, such as Sn, Ge and Bi, can be used instead of Pb in the all-inorganic HPs.^35–37^ Typically, Bi-based HPs have been utilized in photocatalytic and photovoltaic applications due to their optoelectronic properties being comparable with Pb-based ones, including good intrinsic stability and eco-friendliness.^38,39^ For instance, Cs_3_Bi_2_Br_9_ nanocrystals coupled with graphitic carbon nitride (g-C_3_N_4_) nanosheets were found to exhibit a good HGA of 4.6 mmol g^−1^ h^−1^.^40^ Moreover, Pancielejko *et al.*^41^ have demonstrated a 24-fold improvement in HGA by combining Bi-based HPs of Cs_3_Bi_2_X_9_ (X = I, Br, Cl) with different types of TiO_2_. Tang *et al.*^42^ also reported an improved photocatalytic performance for a Cs_3_Bi_2_I_9_/Ti_3_C_2_ composite due to the promoted charge transfer and separation. A stable composite of Cs_3_Bi_2_I_9_ and MoS_2_ quantum dots has also been reported to show great enhancement in photocatalytic performance,^43^ achieving a HGA of 6.09 mmol g^−1^ h^−1^, which notably surpasses that of pristine Cs_3_Bi_2_I_9_ and Pt/Cs_3_Bi_2_I_9_ composite by 8.8 and 2.5 times, respectively. Such an impressive improvement, leading to a new record for Bi-based HP photocatalysts under visible light, was attributed to the type-II heterojunction between Cs_3_Bi_2_I_9_ and MoS_2_.

The experimental findings mentioned above highlight the importance of interface engineering in enhancing the photocatalytic performance of Bi-based HP photocatalysts. To fully understand the underlying mechanism of such an enhancement through interface formation, a first-principles study based on density functional theory (DFT) is necessary.^16^ Since the photocatalytic performance encompasses both the intrinsic properties and the efficiency of a photocatalyst, one should clarify the electronic and optical properties such as band structure, band-edge alignment against the water redox potential, photoabsorption coefficients and the Gibbs free-energy difference upon hydrogen adsorption on the catalysis surface to estimate the photocatalytic performance, which further determines the STH conversion efficiency or HGA. To the best of our knowledge, however, no theoretical studies on the Cs_3_Bi_2_I_9_/MoS_2_ heterostructure have been reported to date, meaning that the effects of interface and defect engineering on its photocatalytic performance remain unclear. In this work, we perform first-principles calculations of the Cs_3_Bi_2_I_9_/MoS_2_ heterostructure to gain atomistic insights into the interfacial and defect effects on the electronic and optical properties. In particular, we consider typical vacancy defects at the interface, evaluating their formation energies, various interfacial properties and HER performance.

## 2 Methods

### 2.1 Computational methods

In the early steps, the DFT calculations were performed by using the pseudopotential plane-wave method as implemented in the Quantum ESPRESSO (QE, version 7.2) package.^44^ For describing the ion–electron interactions, we used the ultrasoft pseudopotentials provided in the GBRV library,^45^ which were generated with scalar relativistic calculations by using the valence electron configurations of Cs-5s^2^5p^6^6s^1^, Bi-6s^2^6p^3^5d^10^, I-5s^2^5p^5^, Mo-4s^2^4p^6^5s^2^4d^4^ and S-3s^2^3p^4^. The Perdew–Burke–Ernzerhof (PBE) formalism^46^ within the generalized gradient approximation (GGA) was used for the exchange–correlation interaction. Since the interface systems under study include layered materials of MoS_2_ and Cs_3_Bi_2_I_9_, the van der Waals (vdW) interactions between the layers were taken into account by using the vdW-DF-ob86 functional.^47^ As the major computational parameters, the kinetic cutoff energies were set to 40 and 400 Ry for wave functions and electron density, respectively, and the special *k*-point meshes for the Brillouin zone sampling were set to (4 × 4 × 2). All the atomic positions were relaxed until the residual force on the atom converged to 5 × 10^−4^ Ry per Bohr, while the crystalline lattice was optimized until the stress was less than 0.05 kbar.

For the supercell calculations to consider the interfacial defects, the pseudo-atomic orbital (PAO) basis sets method as implemented in the SIESTA (version 4.1) package^48^ was used. We selected the double-*ζ* plus polarization (DZP) scheme for the PAO basis sets, which were generated using an energy shift of 300 meV for the orbital-confining cutoff radii and a split norm of 0.30 for the split-valence. The plane-wave cutoff energy was set to 100 Ry and the special *k*-points mesh was set to (4 × 4 × 1) for atomic relaxations and (6 × 6 × 1) for energetic and optical calculations. The atomic relaxations were performed until the atomic forces converged to 0.02 eV Å^−1^. We note that such calculations using the combination of QE and SIESTA packages were found to give reasonable results for different interface systems, with an affordable computational cost.^16,49–51^

The formation energy of a vacancy defect *i* with charge *q* was calculated as follows:^52,53^

1 *E*_f_(*i*,*q*) = *E*_tot_(*i*,*q*) − *E*_perf_ + *μ*_*i*_ + *q*(*E*_F_ + *E*_VBM_),

where *E*_perf_ and *E*_tot_(*i*,*q*) are the DFT total energies of the perfect supercell and the defective supercell containing one vacancy defect *i* with a charge state *q*, *E*_F_ is the Fermi energy referenced to the energy level of the valence band maximum (VBM) *E*_VBM_, and *μ*_*i*_ is the chemical potential of the *i*-th species removed from the supercell. The chemical potential *μ*_*i*_ can be written as *μ*_*i*_ = *E*_*i*_ + Δ*μ*_*i*_, where *E*_*i*_ is the total energy per atom of the corresponding simple substance. Under the thermodynamic equilibrium condition, the existence of bulk Cs_3_Bi_2_I_9_ and MoS_2_ puts constraints on the chemical potentials as follows:^51,54,55^

2 Δ*E*_Cs_3_Bi_2_I_9__ = 3Δ*μ*_Cs_ + 2Δ*μ*_Bi_ + 9Δ*μ*_I_,

3 Δ*E*_MoS_2__ = Δ*μ*_Mo_ + 2Δ*μ*_S_,

where 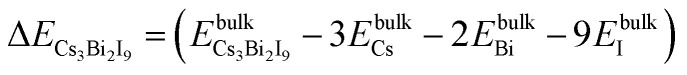 and 
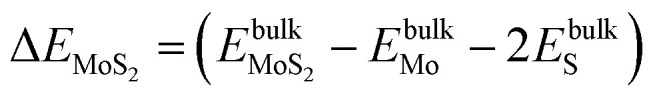
 are the formation energies of the Cs_3_Bi_2_I_9_ and MoS_2_ compounds, respectively. To prevent the formation of elemental bulk *i*, the condition Δ*μ*_*i*_ < 0 should be satisfied. To ensure the formation of Cs_3_Bi_2_I_9_ from the binary compounds CsI and BiI_3_, the following constraints must be met:

4 Δ*μ*_Cs_ + Δ*μ*_I_ < Δ*E*_CsI_

5 Δ*μ*_Bi_ + 3Δ*μ*_I_ < Δ*E*_BiI_3__

For other binary compounds, such as CsI_3_, CsI_4_, Bi_9_I_2_, Cs_3_Bi, CsBi and CsBi_2_, similar constraints are satisfied. Moreover, chemical dissociation of Cs_3_Bi_2_I_9_ into the binary constituents (CsI and BiI_3_) should be prohibited and thus the following condition should be satisfied:

6 Δ*E*_Cs_3_Bi_2_I_9__ − 3Δ*E*_CsI_ < 2Δ*μ*_Bi_ + 6Δ*μ*_I_ < 2Δ*E*_BiI_3__

The hydrogen adsorption energy Δ*E*_H_ is determined as^56^7
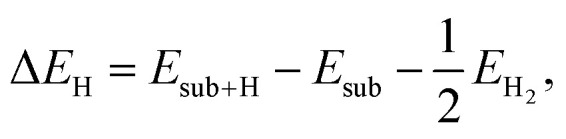
where *E*_sub+H_ and *E*_sub_ are the total energies of the substrate systems with and without an adsorbed H atom on the surface, respectively, and *E*_H_2__ is the total energy of an isolated H_2_ molecule placed in a supercell of the same size as the substrate system, which is large enough to neglect the artificial interactions between the molecule and its periodic images. Then, the Gibbs free energy of hydrogen adsorption Δ*G*_H*_ can be evaluated as follows:

8 Δ*G*_H*_ = Δ*E*_H_ + Δ*E*_ZPE_ − *T*Δ*S*_H_,

where Δ*E*_ZPE_ is the difference between the zero-point energy (ZPE) of H_2_ in the adsorbed state and that in the gas-phase state, and Δ*S*_H_ is the difference in entropy of H_2_ between the gas phase and adsorbed state. Here, Δ*S*_H_ can be written as 
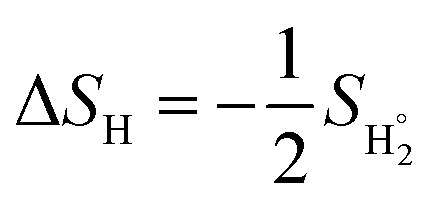
, where 
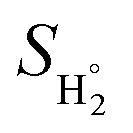
 is the entropy of H_2_ under standard conditions. We utilized 0.24 eV for Δ*E*_ZPE_ − *T*Δ*S*_H_, as reported in the previous work for hydrogen adsorption.^56^

The imaginary and real parts of the frequency-dependent complex dielectric function, *ε*(*ω*) = *ε*_1_(*ω*) + *iε*_2_(*ω*), were calculated using the density functional perturbation theory (DFPT) as implemented in the SIESTA package.^48,57^ Then, the optical absorption coefficients *α*(*ω*) were obtained as follows:^58–60^9
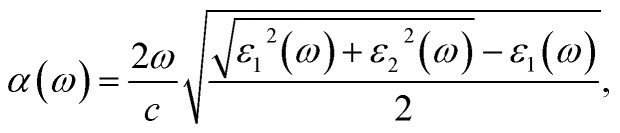
where *c* is the speed of light.

### 2.2 Interface modelling

**Fig. 1:**
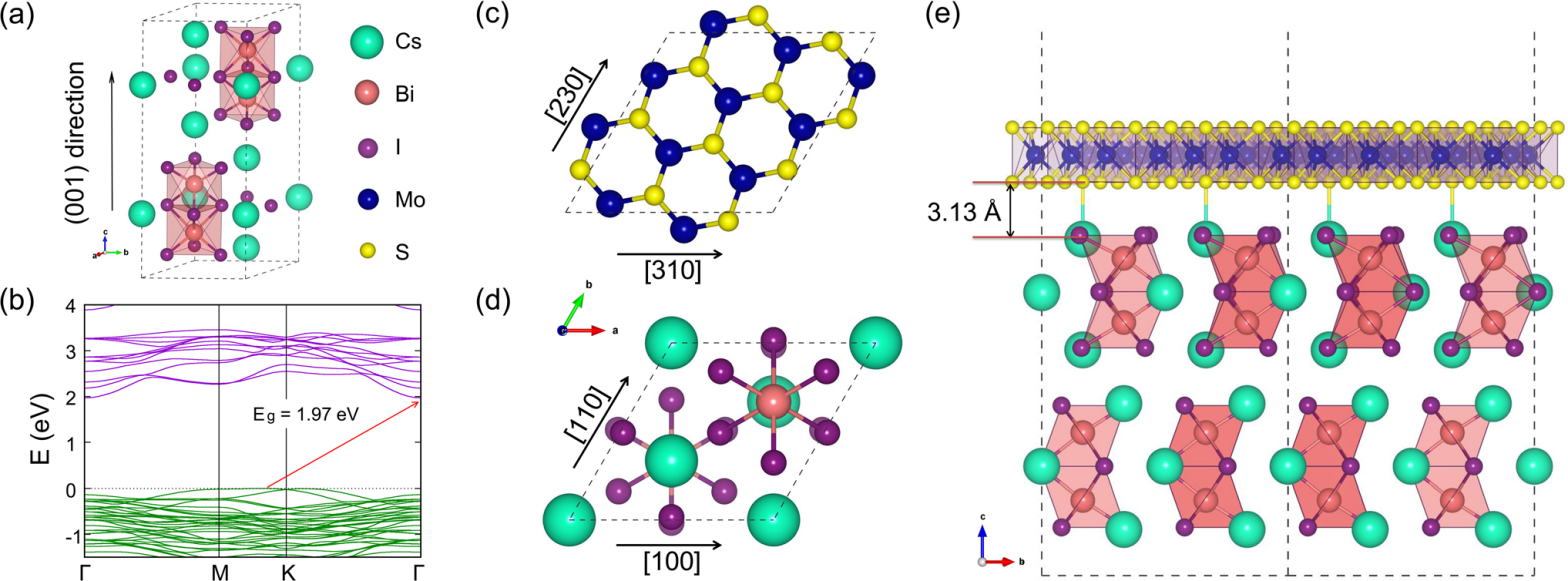
(a) Polyhedral view of the unit cell for the Cs_3_Bi_2_I_9_ crystal optimized with QE. (b) Electronic band structure of the bulk Cs_3_Bi_2_I_9_ calculated in this study. Top view of the lattice-matched structure of (c) the MoS_2_ (001) surface (7 × 7) supercell and (d) the Cs_3_Bi_2_I_9_ (001) surface unit cell. (e) Side view of the Cs_3_Bi_2_I_9_/MoS_2_ ML interface supercell model relaxed with SIESTA.

As a layered vacancy-ordered double perovskite, Cs_3_Bi_2_I_9_ is crystallized in a hexagonal lattice with a space group of *P*6_3_/*mmc* under ambient conditions,^61^ as shown in Fig. 1(a). The structure of Cs_3_Bi_2_I_9_ is characterized by two neighboring [Bi_2_I_9_]^3−^ octahedra, which are face-shared along the *c*-axis to form a [Bi_2_I_9_]^3−^ bioctahedral cluster, and the [Bi_2_I_9_]^3−^ clusters are separated by hexagonal channels filled with Cs^+^ cations, leading to the formation of a zero-dimensional molecular salt crystal structure. Within the [Bi_2_I_9_]^3−^ bioctahedral cluster, there exist three long bridging Bi–I bonds, which involve I atoms on the shared face, and six terminal short Bi–I bonds oriented away from the shared face. For MoS_2_ bulk, we selected the 2H phase among its polymorphs, which is also in a hexagonal lattice with the space group of *P*6_3_/*mmc*. With the optimized bulk structures, we built the interface slab models of Cs_3_Bi_2_I_9_ (001) surface and MoS_2_ (001) monolayer (ML). According to the experimental report for the structural characteristics,^62^ the Cs_3_Bi_2_I_9_ (001) surface was suggested to have CsI_3_ termination. To minimize the lattice mismatch between the Cs_3_Bi_2_I_9_ (001) and MoS_2_ (001) surfaces, we applied the coincidence lattice method.^63^ As a result, we obtained the surface supercells along the lines of [310] and [230] for 2H-MoS_2_ (Fig. 1(c)) and [100] and [110] for Cs_3_Bi_2_I_9_ (Fig. 1(d)), with a significantly small lattice mismatch of 0.1% evaluated as follows:^64^10
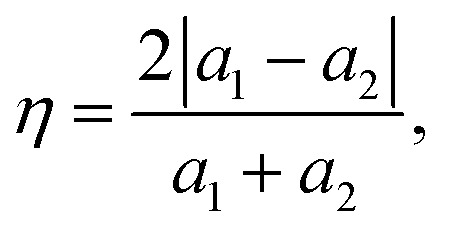
where *a*_1_ and *a*_2_ are the lattice constants of the Cs_3_Bi_2_I_9_ and 2H-MoS_2_ lattices. The resultant supercells correspond to (1 × 1) and (7 × 7) for Cs_3_Bi_2_I_9_ (001) and 2H-MoS_2_ (001) surfaces, respectively. The slab models of the interface contained ten atomic layers of Cs_3_Bi_2_I_9_ and an S–Mo–S layer of MoS_2_ (49 atoms) with a vacuum layer of 15 Å thickness, as shown in Fig. 1(e). We confirm that the 10 atomic layers for the Cs_3_Bi_2_I_9_ is thick enough to guarantee convergence of the surface energy and the 15 Å vacuum thickness is sufficiently large to inhibit the artificial interactions between the top and bottom atoms of the neighbouring slabs. While fixing the lattice constants of the slab supercell and the atomic positions of the 6 atomic layers in the Cs_3_Bi_2_I_9_ side, all other atomic positions were relaxed using the QE code. Here, we considered two different possible sliding structures between Cs_3_Bi_2_I_9_ and MoS_2_, denoted as Conf1 and Conf2 (Fig. S2, SI), and performed atomic relaxations for these sliding configurations. From the calculation, the Conf1 sliding structure, where one of the S atoms in the MoS_2_ layer is placed on top of the Cs atom of the Cs_3_Bi_2_I_9_, was found to have a lower total energy with a shorter interlayer binding distance than the Conf2 structure. Therefore, we only considered the Conf1 sliding structure for further calculations of the electronic and photocatalytic properties.^65^

In order to consider the vacancy defects, we built 2 × 2 × 1 supercells (196 atoms) with a very thick vacuum layer (70 Å) using the relaxed interface slabs with the QE code. The atomic relaxations were also performed using the SIESTA code, while fixing the lattice constants and the 6 atomic layers of the Cs_3_Bi_2_I_9_ side. We confirm that the supercell sizes are large enough for defect calculations as used in the previous works.^16,66^ Then, each of the three types of interfacial vacancy defect, such as caesium (V_Cs_), iodine (V_I_) and sulfur (V_S_), was created at different positions around the interface, and the lowest energy configuration for each defect was identified by performing the atomic relaxation (see Fig. S3 and Table S1, SI). We considered different charge states for each vacancy defect in this work.

The formation energy of the interface is defined as follows:^67^11
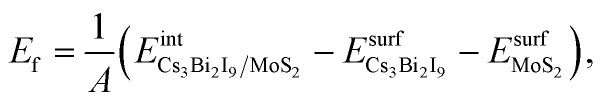
where 
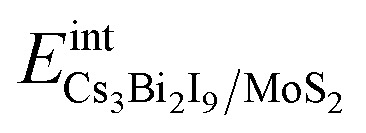
, 
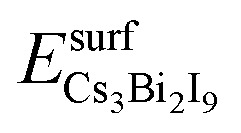
 and 
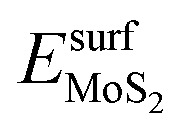
 are the DFT total energies of the Cs_3_Bi_2_I_9_/MoS_2_ interface, the Cs_3_Bi_2_I_9_ (001) surface and the MoS_2_ (001) surface, respectively, and *A* is the interface area. The total energies for the surfaces were calculated by using the fully relaxed isolated surface systems with the same cell size with the interface. The interlayer binding energy is defined as follows:12
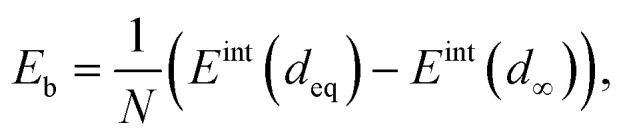
where *E*^int^(*d*_eq_) and *E*^int^(*d*_∞_) are the total energies of the interface systems with the equilibrium interlayer distance and the infinity distance, respectively, and *N* is the number of atoms in the MoS_2_ layer. The spatial charge density difference was evaluated as follows:

13 Δ*ρ* = *ρ*_int_ − *ρ*_Cs_3_Bi_2_I_9__ − *ρ*_MoS_2__,

where *ρ*_int_, *ρ*_Cs_3_Bi_2_I_9__ and *ρ*_MoS_2__ are the charge densities of the interface, isolated Cs_3_Bi_2_I_9_ and MoS_2_ surface sides, respectively. Then, the planar-averaged charge density difference integrated on the *x* − *y* plane was calculated as follows:14
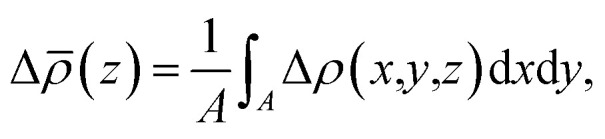
where *A* is the area of the *x* − *y* plane.^16,51^

## 3 Results and discussion

### 3.1 Structural stability of the interface

Firstly, we scrutinized the geometrical and electronic properties of bulk Cs_3_Bi_2_I_9_ and 2H-MoS_2_. By performing the structural optimizations of the unit cells, we determined their lattice constants to be *a* = *b* = 8.371 Å, *c* = 21.132 Å for Cs_3_Bi_2_I_9_ and *a* = *b* = 3.167 Å, *c* = 12.374 Å for 2H-MoS_2_, which agree well with the previous experimental results.^61,68,69^ Using the QE code with the PBE functional, we then calculated their electronic band structures. Fig. 1(b) shows the calculated band structure of Cs_3_Bi_2_I_9_, demonstrating an indirect bandgap of 1.97 eV in good agreement with the previous results.^61,70^ Meanwhile, the band structure of the 2H-MoS_2_ monolayer shows a direct bandgap of 1.84 eV, also in good agreement with the previous DFT calculations^69^ (see Fig. S1, SI). Such good agreements indicate that the computational settings employed in this work are reasonably acceptable. We note that the GGA-PBE functional was found to give bandgap values that agreed well with the experimental ones for lead halide perovskites, due to the fortuitous error compensation between the underestimation of PBE and overestimation by ignoring the spin–orbit coupling effect.^71,72^

With these calculated lattice constants of the bulk materials, the cell dimensions of the Cs_3_Bi_2_I_9_/MoS_2_ interface were found to be 8.375 × 8.375 Å and those of the 2 × 2 supercells was 16.75 × 16.75 Å. To estimate the stability of the Cs_3_Bi_2_I_9_/MoS_2_ interface system, the formation energy *E*_f_ was calculated. The formation energy was found to be negative, indicating that the formation of the Cs_3_Bi_2_I_9_/MoS_2_ interface is exothermic and that binding between the constituent Cs_3_Bi_2_I_9_ surface and MoS_2_ monolayer is energetically favorable. In fact, the formation energy of the perfect heterostructure without any defects was calculated to be −0.70 J m^−2^, being lower than that of the CsPbI_3_/MoS_2_ interface obtained in our previous work^16^ (−0.22 and −0.37 J m^−2^ for CsI and PbI_2_ terminations, respectively). This indicates the stronger binding between the constituent layers in the Cs_3_Bi_2_I_9_/MoS_2_ heterostructure than in the CsPbI_3_/MoS_2_ heterostructure.

**Fig. 2:**
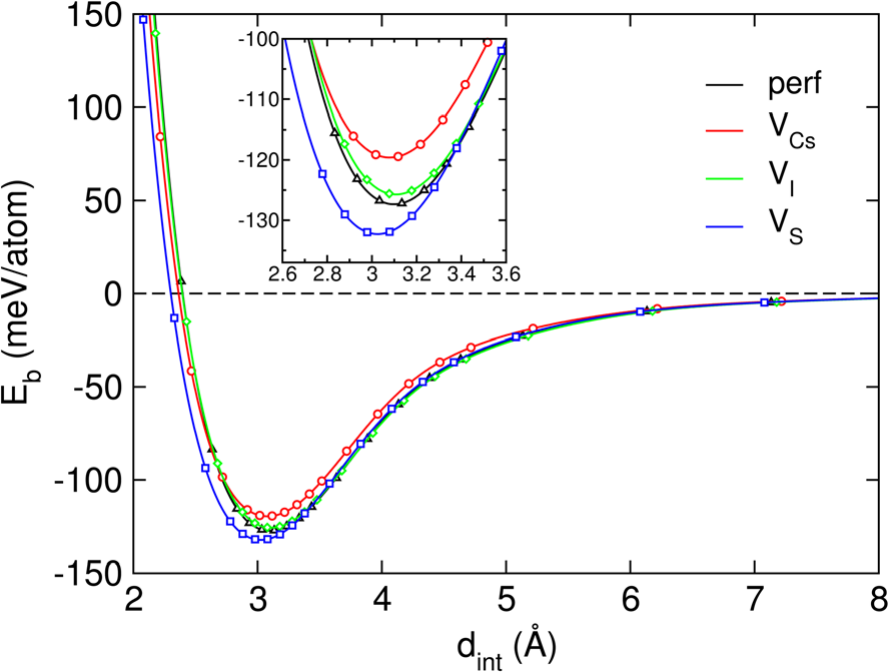
The interlayer binding energy per atom *E*_b_ for the Cs_3_Bi_2_I_9_/MoS_2_ heterojunction as a function of interlayer binding distance *d*_int_. The inset represents the magnified view around the equilibrium distance *d*_eq_.

To further assess the binding strength between the constituent layers of the interface, the interlayer binding energy was also calculated. Fig. 2 shows the resultant *E*_b_ as a function of interlayer distance *d*_int_. Table 1 lists the calculated values for the interface formation energy, interlayer binding energies and interlayer equilibrium distances for the Cs_3_Bi_2_I_9_/MoS_2_ heterostructures considered in this work. Irrelevant to the existence of vacancy defects, the binding energy *E*_b_ values for the interface systems were found to be negative, indicating the attractive binding in the Cs_3_Bi_2_I_9_/MoS_2_ interface systems. The binding energy for the perfect system was estimated to be −127.81 meV per atom, which is comparable to that of the CsPbI_3_/MoS_2_ interface.^16,73^ Among the different defective systems, the V_S_ interface system was found to have the lowest binding energy of −132.54 meV per atom and accordingly the shortest interlayer distance of 3.08 Å. For the cases of V_I_ and V_Cs_, the *E*_b_ values were found to slightly increase to −125.81 and −117.95 meV per atom, while the *d*_int_ values decrease to 3.18 and 3.22 Å, respectively.

**Table 1:** The interface formation energy *E*_f_, interlayer binding energy *E*_b_ and interlayer equilibrium distances *d*_int_ in the Cs_3_Bi_2_I_9_/MoS_2_ heterostructures without and with vacancy defects

System	*E*_f_ (J m^−2^)	*E*_b_ (meV per atom)	*d*_int_ (Å)
Perfect	−0.70	−127.82	3.13
V_Cs_		−117.95	3.22
V_I_		−125.81	3.18
V_S_		−132.54	3.08

### 3.2 Formation of interfacial vacancy defect

Defects play a decisive role in the photovoltaic and photocatalytic applications. To check the formation possibility of the interfacial vacancy defects under the synthesis conditions, we estimated their formation energies from the thermodynamic point of view. According to eqn (1), the defect formation energy varies with the chemical potentials of the elements, which reflect the synthesis conditions in the experiments. Therefore, we first determined the chemical potential ranges of all the species included in the interface system for ensuring the stable existence of bulk Cs_3_Bi_2_I_9_ and MoS_2_ by using the thermodynamic constraints given by eqn (2)–(6) and the formation energy values of the available compounds (see Table S2 and Fig. S4, SI). Note that none of the ternary compounds are available in the Cs-Bi-I system, except for Cs_3_Bi_2_I_9_.

**Fig. 3:**
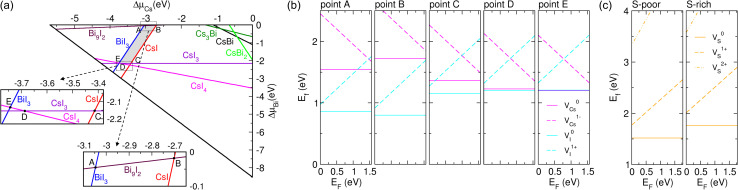
(a) Chemical potential ranges of Cs and Bi for the stable Cs_3_Bi_2_I_9_ compound, marked as grey-colored pentagon ABCDE. Outside the pentagon, the other binary compounds such as CsI, BiI_3_, CsI_3_, CsI_4_, Bi_9_I_2_, CsBi, Cs_3_Bi and CsBi_2_ will form instead of the Cs_3_Bi_2_I_9_ perovskite. Insets depict the magnified views around the vertexes of the pentagon. (b) Calculated formation energies of V_Cs_ and V_I_ defects at theCs_3_Bi_2_I_9_ part of the interface as a function of the Fermi energy (*E*_F_) at points A, B, C, D and E. (c) The formation energy of vacancy defect V_S_ at the MoS_2_ part of the interface under S-poor and S-rich conditions.

Fig. 3(a) presents the chemical potential diagram obtained in this work concerning the chemical potentials of Cs (Δ*μ*_Cs_) and Bi (Δ*μ*_Bi_), which are referenced to those of the corresponding elemental bulks. The thermodynamically stable region for the Cs_3_Bi_2_I_9_ compound is marked as the grey-colored region shown as the pentagon ABCDE (see Table S3 for the chemical potential values at the five vertexes of the pentagon, ESI). Among the vertex points of the pentagon, point B has the maximum value of Δ*μ*_Cs_ and the minimum value of Δ*μ*_I_ (Δ*μ*_Cs_ = −2.70 eV, Δ*μ*_Bi_ = 0.02 eV, Δ*μ*_I_ = −0.99 eV), while point E has the maximum value of Δ*μ*_I_ and the minimum value of Δ*μ*_Cs_ (Δ*μ*_Cs_ = −3.74 eV, Δ*μ*_Bi_ = −2.13 eV, Δ*μ*_I_ = −0.17 eV). Therefore, point B is said to represent the Cs-rich/I-poor condition, while point E represents the I-rich/Cs-poor condition. The possible chemical potential ranges for Mo and S species were also evaluated (see Table S3, SI). Note that outside of the pentagon region, the undesirable binary compounds such as CsI, BiI_3_, CsI_3_ and CsI_4_ are more stable than Cs_3_Bi_2_I_9_. For instance, BiI_3_ will form in the left-hand side of the A–E line, while the CsI compound is stable in the right-hand side of the B–C line. It is worth noting that the stable region has a rather wide shape in contrast to the very narrow shapes for the Pb-based HPs, such as MAPbI_3_ and CsPbI_3_.^16,51,73^ This indicates that the Bi-based HP Cs_3_Bi_2_I_9_ is more stable than the Pb-based HPs, for which instability is the main obstacle to their photovoltaic applications.

We then calculated the band structures of the Cs_3_Bi_2_I_9_ and MoS_2_ surfaces having the same planar size as the interface to determine the Fermi levels *E*_F_, bandgaps 
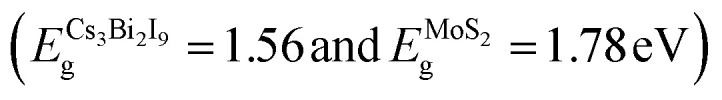
 and VBM levels (*E*_VBM_) (see Fig. S5, SI). To determine the *E*_VBM_ levels for the interface, we adopted the line-up-averaged electrostatic potential method^74,75^ for the Cs_3_Bi_2_I_9_/MoS_2_ interface, and Cs_3_Bi_2_I_9_ and MoS_2_ surfaces (see Fig. S6, SI). From the obtained band structures and the work functions (calculated by *ϕ* = *E*_v_ − *E*_F_, where *E*_v_ is the vacuum level), the VBM levels were corrected to be 
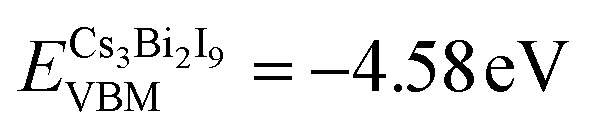
 and 
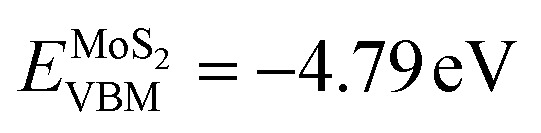
 for the Cs_3_Bi_2_I_9_ and MoS_2_ sides, respectively.

Fig. 3(b) shows the calculated formation energies of the vacancy defects V_Cs_ and V_I_ as functions of *E*_F_ at the points A, B, C, D and E. The formation energies of all the vacancy defects were found to be positive, indicating that their formation is endothermic. In most cases (points A, B, C, and D), V^0^_I_ has the lowest formation energy, suggesting that V^0^_I_ is easier to form than other vacancies and thus the dominant defect at the Cs_3_Bi_2_I_9_/MoS_2_ interface. In particular, this argument is more evident under the I-poor/Cs-rich condition (points A, B and C). Note that in the Pb-based HPs, the V_I_ defect was also found to be the major defect with the lowest formation energy.^54,55^ When going to the I-rich/Cs-poor condition (points D and E), it is natural to observe that the formation energy of V_Cs_ decreases while that of V_I_ increases, and finally V_Cs_ has a lower formation energy than V_I_ at point E. On the other hand, V_S_ was also found to have a positive formation energy, as shown in Fig. 3(c), implying that its formation at the interface is not spontaneous but endothermic in accordance with the case of the MoS_2_ bulk.^76^ Moreover, the formation energy of the V^0^_S_ state was found to be lower than those of the V_S_^1+^ and V_S_^2+^ states in the whole range of *E*_F_. Interestingly, none of the thermodynamic transition levels between the differently charged defects, where the electrons could be donated or accepted, were observed, suggesting that these vacancy defects do not cause the undesirable charge recombination at the Cs_3_Bi_2_I_9_/MoS_2_ interface.

### 3.3 Interfacial charge redistribution and DOS analysis

**Fig. 4:**
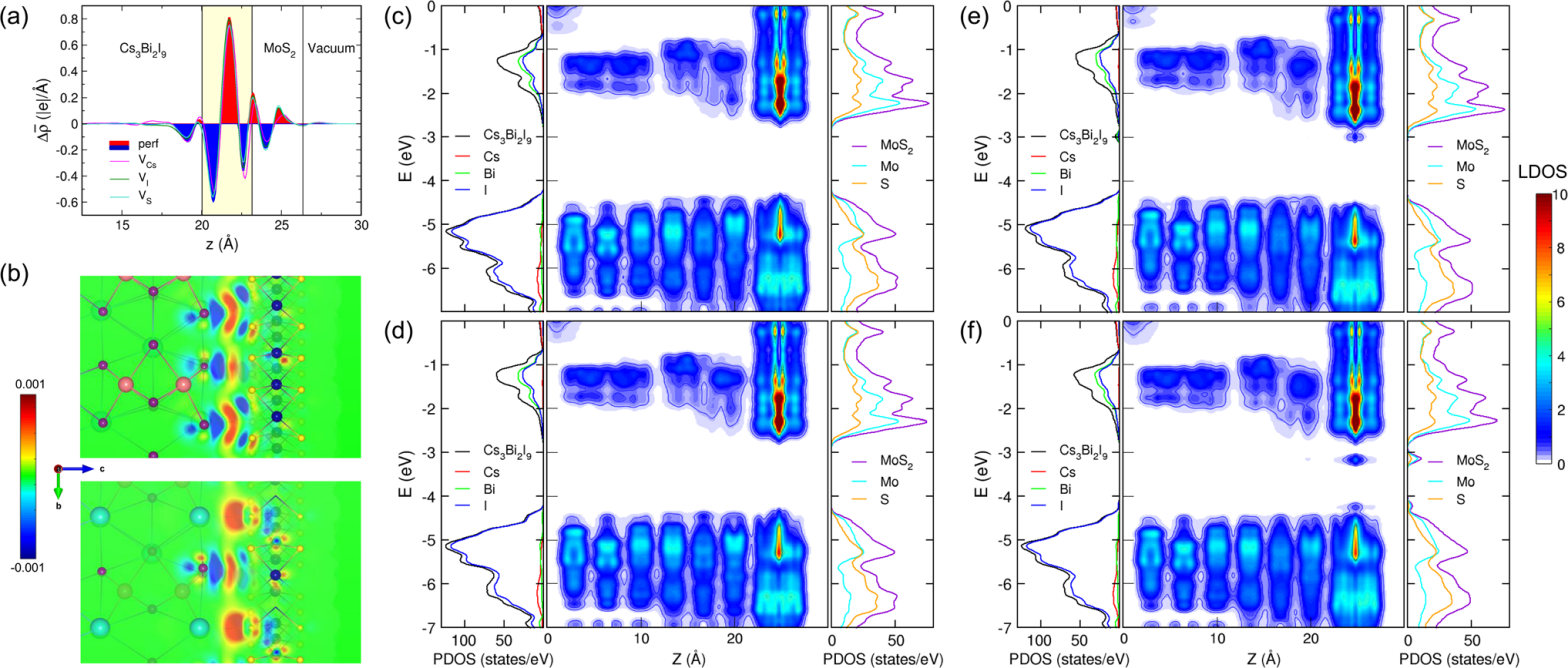
(a) Planar-averaged charge density difference (Δ*ρ̄*) integrated over the *x*–*y* plane along the *z*-axis upon the formation of Cs_3_Bi_2_I_9_/MoS_2_ interfaces without and with the vacancy defects under study. (b) Isosurface plot of the spatial charge density difference upon the formation of the perfect interface projected on the (100) planes passing the I atoms (top panel) and Cs atoms (bottom panel) of the Cs_3_Bi_2_I_9_ surface, ranging from 0.001 to 0.001 |e| Å^−3^. Partial density of states (PDOS) and isosurface and isoline plots of local density of states (LDOS) for (c) the perfect interface, (d) V_Cs_-, (e) V_I_- and (f) V_S_-containing interfaces.

We evaluated the charge density difference upon interface formation to gain intuitive insights into the binding strength and charge carrier transfer across the interface. Fig. 4(a) depicts the calculated planar-averaged charge density differences upon the formation of Cs_3_Bi_2_I_9_/MoS_2_ interfaces without and with the vacancy defects. At a glance, the charge redistribution occurs locally around the interface region, while being barely influenced by the defect formation. The charge accumulation was observed mostly in the interstitial region biased to the MoS_2_ side, whereas the charge depletion was found mostly around the CsI_3_ layer of the Cs_3_Bi_2_I_9_ side. This observation is further clarified by the isosurface plot of the spatial electron density difference, as can be seen in Fig. 4(b), where the charge depletion (blue colour) is distributed around the I atoms of Cs_3_Bi_2_I_9_, while charge accumulation (red colour) is seen around the S atoms of the MoS_2_ layer (see Fig. S7 for the defective systems, SI). The observed charge redistribution indicates that the significant amount of electrons are transferred from the MoS_2_ layer to the Cs_3_Bi_2_I_9_ side, leading to the strong binding between them at the interface. Moreover, the interface dipole originating from this charge redistribution is expected to help the transfer of photogenerated electrons and holes as will be discussed later.

For a quantitative insight into the charge redistribution, we integrated the planar-averaged electron density difference to obtain the total transferred charge as Δ*q* = ∫Δ*ρ̄*(*z*)d*z*, which was further divided into the contributions from the Cs_3_Bi_2_I_9_ (Δ*q*_Cs_3_Bi_2_I_9__) and MoS_2_ (Δ*q*_MoS_2__) sides. Table 2 lists the calculated transferred charge contributions from two constituent layers in the perfect and defective interfaces. For all the cases, the Cs_3_Bi_2_I_9_ part was found to have negative values whereas the MoS_2_ side had positive ones. This indicates that charge is depleted at the terminal surface of Cs_3_Bi_2_I_9_, while it is accumulated at the MoS_2_ monolayer, resulting in the creation of an interfacial dipole moment oriented from the Cs_3_Bi_2_I_9_ side to the MoS_2_ side, which prevents the charge carriers from transferring across the interface. Among the different systems without and with the vacancy defects, the largest charge transfer was observed in the V_I_-containing system, whereas the smallest charge transfer occurred in the V_Cs_-containing interface.

**Table 2:** Transferred charge upon the formation of the Cs_3_Bi_2_I_9_/MoS_2_ heterostructures under study, which is divided into contributions from each of the subsystems (Δ*q*_Cs_3_Bi_2_I_9__, Δ*q*_MoS_2__), and the interfacial dipole moment Δ*μ*

System	Δ*q*_Cs_3_Bi_2_I_9__ (|e|)	Δ*q*_MoS_2__ (|e|)	Δ*μ* (Debye)
Perfect	−0.306	0.308	4.23
V_Cs_	−0.202	0.204	2.41
V_I_	−0.318	0.321	4.79
V_S_	−0.250	0.254	4.29

Charge transfer often induces interface polarization, which plays an important role in the properties of a heterostructure and is quantified by the interfacial dipole moment. As mentioned above, the interfacial dipole moment, orienting from the Cs_3_Bi_2_I_9_ side to the MoS_2_ side, helps the photogenerated electrons and holes move across the interface in opposite directions, thereby being favourable for preventing the recombination of photogenerated charge carriers and enhancing the photocatalytic performance in the Cs_3_Bi_2_I_9_/MoS_2_ heterostructure. The interface polarization induces a step in the electrostatic potential across the interface, which results in changes in the band-edge alignment. The interfacial dipole moment also originates the downward and upward band bending with the formation of a charge accumulation layer at the interface. The interfacial dipole moment, induced at the interface from the charge redistribution, was evaluated by Δ*μ* = −∫*z*Δ*ρ̄*d*z*. The calculated Δ*μ* values are listed in Table 2. The V_I_-containing interface was also found to have the largest Δ*μ* value of 4.79 D among the interface systems under study. Meanwhile, the V_Cs_ defect causes a reduction in Δ*μ* to 2.41 D. Such tendencies are consistent with the previous calculations for CsPbI_3_/2H-MoS_2_ (ref. 16) and MAPbI_3_/2H-MoS_2_ interfaces,^77^ where V_I_ defect systems have obviously larger Δ*μ* values and V_Cs_ systems have smaller Δ*μ* values. When compared with those of the Pb-based HP/MoS_2_ heterostructures, however, Δ*μ* values are relatively larger, suggesting further improvement of photocatalytic performance in the Cs_3_Bi_2_I_9_/MoS_2_ interface.

In order to understand the generation and transport processes of charge carriers induced by photon incidence, we calculated the electronic density of states (DOS) of the interface systems. Fig. 4(c)–(f) display the calculated atom-projected partial density of states (PDOS) of the Cs_3_Bi_2_I_9_ and MoS_2_ sides and the local density of states (LDOS) along the *z*-axis for all the interface systems. Our calculations revealed that the lower conduction bands (CBs) responsible for the transport of photo-induced electrons are contributed from Bi and I atoms on the Cs_3_Bi_2_I_9_ side and both Mo and S atoms on the MoS_2_ side. From the LDOS plots, the CB edge states of MoS_2_ were found to be lower in energy than those of Cs_3_Bi_2_I_9_, being beneficial to the transfer of electrons photo-generated in the Cs_3_Bi_2_I_9_ side to the MoS_2_ side. It was found that the V_Cs_ defect causes barely any change in the electronic properties of the perfect interface system, whereas the V_I_ and V_S_ defects induce shallow trap states near the CBM level. In particular, the V_S_-containing interface exhibits the trap state relatively far below the CBM level. Since trap states inside the bandgap would behave as recombination centers, they can be detrimental to the photocatalytic performance. However, it can be said that the V_S_ defect is difficult to be formed due to its relatively high formation energy.

### 3.4 Photocatalytic performance

In understanding the photocatalytic performance for HER, the band edge alignment is an important criterion. For a high HER performance, it is required that the CBM level should be higher than the hydrogen reduction potential of H^+^/H_2_, while the VBM level should be lower than the water oxidation potential of O_2_/H_2_O. Selecting the vacuum level as the reference, the hydrogen reduction potential and the water oxidation potential are calculated at different pH values as follows:^56,73^15
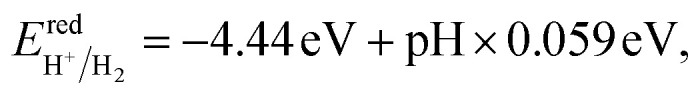
16
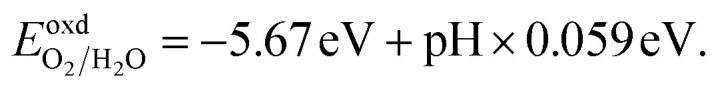


Once the Cs_3_Bi_2_I_9_ surface is combined with the MoS_2_ monolayer to build the heterostructure, some electrons are transferred to ensure the same Fermi level at the interface.^64^ By using the calculated work function *ϕ*, we determined the Fermi levels of the Cs_3_Bi_2_I_9_/MoS_2_ interfaces, and then obtained the *E*_VBM_ and *E*_CBM_ values for the Cs_3_Bi_2_I_9_ and MoS_2_ parts, which are referenced to the vacuum level *E*_v_ (see Fig. S8 for the band structures, SI).

**Fig. 5:**
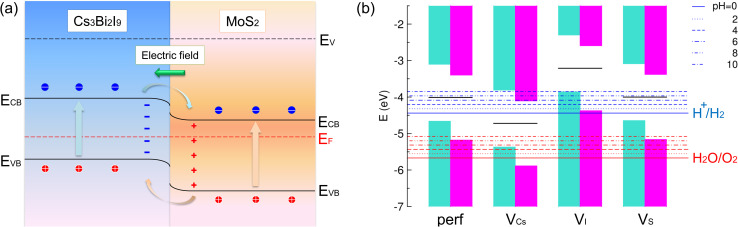
(a) Schematic diagram of band alignment in the Cs_3_Bi_2_I_9_/MoS_2_ interface with a type-II heterojunction. Here, *E*_v_ and *E*_F_ are the vacuum and Fermi energy levels, and *E*_VB_ and *E*_CB_ are the VBM and CBM levels of the Cs_3_Bi_2_I_9_ and MoS_2_ sides. (b) Band-edge alignments of Cs_3_Bi_2_I_9_/MoS_2_ interface systems, referenced to the vacuum level. Turquoise and magenta bars indicate VBs and CBs of the Cs_3_Bi_2_I_9_ and MoS_2_ parts, respectively. The Fermi level is depicted as a horizontal black line for each interface system. Blue and red horizontal long lines represent the hydrogen reduction and water oxidation potentials, respectively, at different pH values.

For the perfect interface system, the VBM and CBM levels were calculated to be −4.66 and −3.10 eV for the Cs_3_Bi_2_I_9_ side and −5.18 and −3.40 eV for the MoS_2_ side, respectively. Therefore, the interface was characterized as a type-II heterojunction, being significantly beneficial to the separation of electrons and holes in opposite directions, *i.e.*, preventing the recombination of charge carriers. The interfacial dipole moment induced from the charge redistribution originates the downward and upward band bending with the formation of a charge accumulation layer at the interface.^75^Fig. 5(a) shows a scheme that illustrates the generation and transfer processes of charge carriers at the heterojunction. When photons are absorbed in the Cs_3_Bi_2_I_9_/MoS_2_ heterojunction, electrons are excited from the VBM to the CBM on each side of the interface, due to their proper bandgaps for light absorption. Then, the electrons placed on the CBM of Cs_3_Bi_2_I_9_ are attracted toward the MoS_2_ side by the interface dipole, while the holes left in the VBM of MoS_2_ are drawn to the Cs_3_Bi_2_I_9_ side.

Fig. 5(b) illustrates the calculated band-edge alignments of the interface systems under study (see Table S4 for the corresponding numerical values, SI). In all cases, the CBM is higher than the H^+^/H_2_ reduction potential, indicating that the Cs_3_Bi_2_I_9_/MoS_2_ heterostructure has the potential to reduce H^+^ to H_2_. In the case of the V_I_-containing interface, however, it was revealed that the band-edge alignment is not appropriate for water splitting, since the VBM is even higher than the H^+^/H_2_ potential. For the perfect interface and V_S_-defective systems, the CBMs are lower than the O_2_/H_2_O potential only at pH values higher than 8, *i.e.*, in the basic environment. Therefore, they cannot produce O_2_ by oxidizing H_2_O under acidic conditions. With the proper band-edge alignment, the V_Cs_-containing interface is likely to be the most promising photocatalyst for water redox reactions, especially in an acidic environment.

**Fig. 6:**
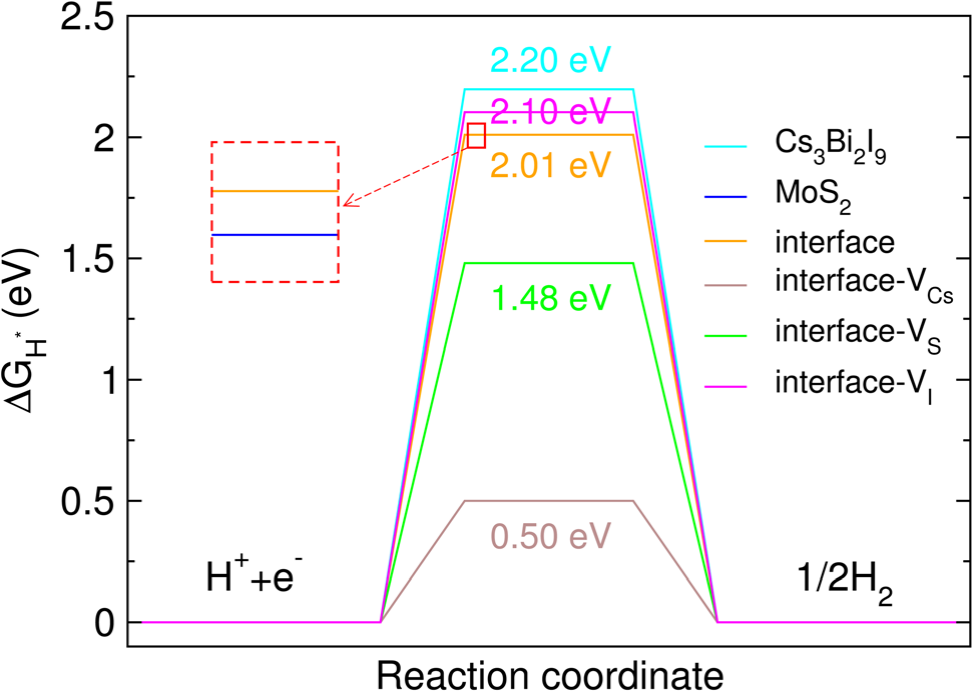
Gibbs free energy difference for hydrogen adsorption (Δ*G*_H*_) on the Cs_3_Bi_2_I_9_/MoS_2_ interface systems without and with the vacancy defects such as V_Cs_, V_I_ and V_S_, together with those on the pristine Cs_3_Bi_2_I_9_ surface and MoS_2_ monolayer.

For further insights into the photocatalytic performance of the interface, we evaluated the Gibbs free energy for hydrogen adsorption (Δ*G*_H*_).^56^ From the viewpoint of HER activity, the absolute value of Δ*G*_H*_ should be as small as possible. We calculated Δ*G*_H*_ for the Cs_3_BI_2_I_9_/MoS_2_ heterojunctions, together with the isolated Cs_3_BI_2_I_9_ surface and the MoS_2_ monolayer. Considering the previous result,^16^ we proposed that the H atom could be adsorbed on the S atom in the top layer of the MoS_2_ monolayer (see Fig. S9, SI). For the case of the Cs_3_Bi_2_I_9_ surface, the adsorption site of the H atom was identified using energetic calculations. As shown in Fig. 6, the Cs_3_Bi_2_I_9_ surface was found to show the highest Δ*G*_H*_ value of 2.20 eV, while the perfect heterostructure and MoS_2_ monolayer have nearly the same value of 2.01 eV, which is consistent with the Δ*G*_H*_ value obtained for the graphene/MoS_2_ heterostructure.^78^ This indicates that the formation of the interface between Cs_3_Bi_2_I_9_ and MoS_2_ without any defects hardly affects on the Gibbs free energy for hydrogen adsorption on the MoS_2_ layer. The vacancy defect formation at the interface was found to have a positive effect on HER activity due to the lower Δ*G*_H*_, except for the V_I_ defect which was found to slightly increase the Δ*G*_H*_ value. For V_S_- and V_Cs_-containing systems, Δ*G*_H*_ was reduced to 1.48 and 0.50 eV, respectively, implying some improvement of HER performance.

**Fig. 7:**
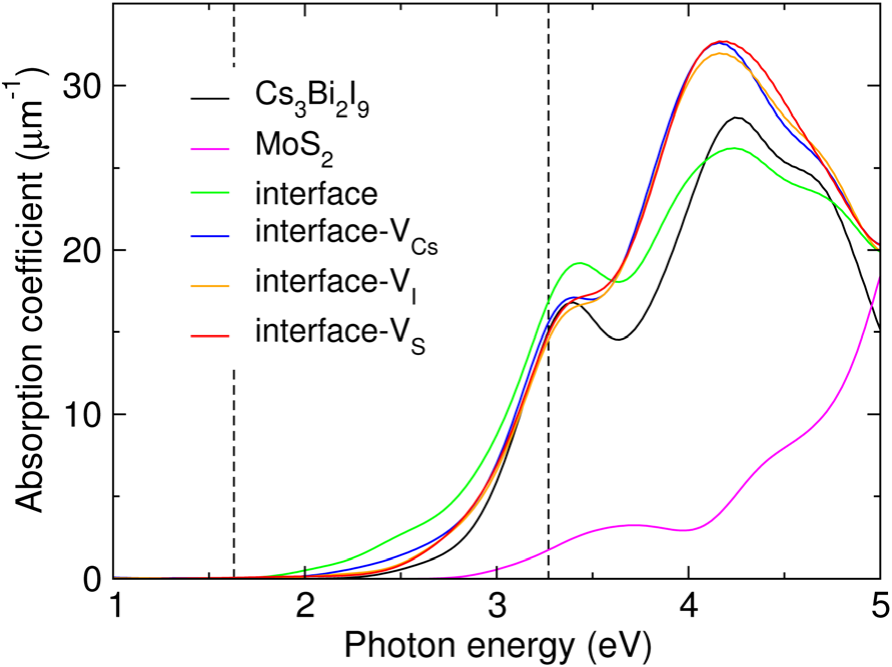
Photoabsorption spectra of bulk Cs_3_Bi_2_I_9_, the MoS_2_ monolayer and the Cs_3_Bi_2_I_9_/MoS_2_ interface systems without and with a vacancy defect. The vertical dashed lines indicate the visible-light region.

For the final step, we calculated the photoabsorption spectra of the Cs_3_Bi_2_I_9_/MoS_2_ heterostructures without and with the interfacial vacancy defect, bulk Cs_3_Bi_2_I_9_ and the MoS_2_ monolayer, as shown in Fig. 7. From these spectra, we could estimate the photoabsorption edge and intensity, which are crucial factors to achieve high photocatalytic activity (see Fig. S10 for dielectric functions and other optical properties, SI). For bulk Cs_3_Bi_2_I_9_ and the MoS_2_ monolayer, the absorption edges were found to be 1.54 and 1.84 eV, respectively. Meanwhile, those of the heterostructures were found to be significantly shifted to the lower-energy region varying from 1.18 to 1.48 eV. Moreover, the Cs_3_Bi_2_I_9_/MoS_2_ heterostructures exhibited higher absorption intensities than their constituents in the visible-light and near-ultraviolet regions, representing their superior photoabsorption capability. The perfect interface has greater absorption intensity than the defective interfaces in the visible-light region, while in the ultraviolet region the defective heterostructures have higher absorption coefficients than the perfect interface. It is worth noting that among the different vacancy defects, the V_Cs_ defect induces the greatest intensity in the visible-light region. This is mainly due to the reduced bandgaps and the built-in electric field originating from the charge redistribution at the interface.^79,80^

## 4 Conclusions

We have systematically investigated the structural, electronic and optical properties of Cs_3_Bi_2_I_9_/MoS_2_ heterostructures using first-principles calculations to give a comprehensive theoretical understanding on the enhancement of photocatalytic water splitting activity for hydrogen evolution. Using the (1 × 1) unit cell of the Cs_3_Bi_2_I_9_ (001) surface and a (7 × 7) cell of the 2H-MoS_2_ monolayer, we built a slab model of the Cs_3_Bi_2_I_9_/MoS_2_ interface with a very small lattice mismatch of 0.1% without and with a vacancy defect, such as V_Cs_, V_I_ and V_S_. The formation and binding energies of the interface systems were calculated, confirming that these heterostructures are energetically stable. We computed the defect formation energies in the defective Cs_3_Bi_2_I_9_/MoS_2_ interfaces under the different growth conditions through chemical potentials of the species, finding that the V_I_ defect could be generated with the lowest formation energy under the I-poor/Cs-rich condition, while the V_Cs_ defect would be dominant under the Cs-poor/I-rich condition. Through the analysis of charge redistribution upon interface formation, we demonstrated that some electrons are transferred from the MoS_2_ side to the Cs_3_Bi_2_I_9_ side, inducing the interfacial dipole moment beneficial to preventing the recombination of photogenerated charge carriers. Our calculations of DOS and photoabsorption spectra revealed that the perfect interface system and the V_Cs_-containing interface could be favourable for photocatalytic activity compared with other defective systems, since they do not induce shallow trap states inside the bandgap range and exhibit higher absorption intensities than others. Finally, we found that the V_Cs_-containing heterostructure is particularly promising for improving the photocatalytic activity for hydrogen generation due to the significant reduction of the Gibbs free energy for hydrogen adsorption to 0.5 eV and the most suitable band alignment for photo-induced overall water splitting, whose band edge positions straddle the water redox potentials.

## Author contributions

Yun-Sim Kim and Chol-Jun Yu developed the original project. Kyong-Mi Kim and Yun-Sim Kim performed the calculations and drafted the first manuscript. Dok-Ho Hyon and Chol-Hyok Ri contributed to useful discussions. Chol-Jun Yu supervised the work. All authors reviewed the manuscript.

## Conflicts of interest

There are no conflicts to declare.

## Supplementary Material

RA-015-D5RA05294G-s001

## Data Availability

The data are available in the supplementary information (SI) and further from the authors upon reasonable request. Supplementary information: Tables for defect position search, formation energies of the compounds, chemical potential values and band alignment, and figures for the band structure of the MoS_2_ monolayer, two different sliding configurations, vacancy sites at the interfaces, the Cs-Bi-I ternary system, band structures and electrostatic potential of the interface systems, spatial charge density difference of the heterostructures with vacancy defects, adsorption geometries for hydrogen adsorption on different systems, real and imaginary parts of the dielectric function and other optical properties. See DOI: https://doi.org/10.1039/d5ra05294g.
