# Supplementary material for: First-principles study on enhancing the photocatalytic hydrogen evolution performance in Cs3Bi2I9/MoS2 heterostructure with interfacial defect engineering
Source: RSC Adv. 2025 Oct 2;15(43):36607–17. doi: 10.1039/d5ra05294g (PMC12489549; doi:10.1039/d5ra05294g)
Supplement: RA-015-D5RA05294G-s001 [file RA-015-D5RA05294G-s001.pdf]

Supplementary Information (SI) for RSC Advances.
This journal is © The Royal Society of Chemistry 2025

Electronic Supplementary Information – First-principles study on enhancing the photocatalytic hydrogen evolution performance in $Cs_3Bi_2I_9/MoS_2$ heterostructure with interfacial defect engineering

Kyong-Mi Kim[a], Yun-Sim Kim[a], Dok-Ho Hyon[b], Chol-Hyok Ri[a,c] and Chol-Jun Yu[a*]

[a] *Computational Materials Design, Faculty of Materials Science, Kim Il Sung University, Taesong District, Pyongyang, Democratic People's Republic of Korea*

[b] *Institute of Nano Engineering, State Academy of Science, Rakrang District, Pyongyang, Democratic Peoples Republic of Korea*

[c] *Faculty of Physics, O Jung Hup Chongjin University of Education, Chongjin, North Hamgyong Province, Democratic People's Republic of Korea*

Table S1. Total energies calculated for $Cs_3Bi_2I_9/MoS_2$ heterostructures with all possible interfacial vacancies. The lowest energy for each vacancy defect is highlighted.

Vacancy	Number	Energy (eV)
V_{Cs}	1	**−48883.7065**
V_I	1	−48663.9579
	2	−48663.7683
	3	**−48663.9663**
V_S	1	−48699.4159
	2	**−48699.4169**
	3	−48699.2575
	4	−48699.4156
	5	−48699.2868
	6	−48699.2615
	7	−48699.2594

*Corresponding author: Chol-Jun Yu, Email: cj.yu@ryongnamsan.edu.kp

Table S2. The space group and the formation energies of the compounds used for determining the chemical potentials of each specie.

Solid	Space group	Ref	Formation energy (eV)
CsI	$Pm\bar{3}m$ (cubic)	[1]	−3.692
CsI_3	*Pmcn* (orthorhombic)	[2]	−4.248
Cs_2I_8	$P12_1/a1$ (monoclinic)	[3]	−4.435
CsBi	$P12_1/c1$ (monoclinic)	[4]	−1.100
Cs_3Bi	$Fm\bar{3}m$ (cubic)	[5]	1.960
$CsBi_2$	$Fd\bar{3}ms$ (cubic)	[6]	−1.317
BiI	$C12m1$ (monoclinic)	[7]	−0.904
BiI_3	$P3\bar{1}m$ (rhombohedral)	[8]	−2.652
Bi_9I_2	$P21/m$ (monoclinic)	[9]	−2.145
$Cs_3Bi_2I_9$	$P6_3/mmc$ (hexagonal)	[10]	−17.052
MoS_2	$P63mmc$ (hexagonal)	[11]	−2.898

Table S3. Chemical potential values for Cs, Bi and I atoms at point A, B, C, D and E, and those for Mo and S atoms in S-poor and S-rich conditions.

$Cs_3Bi_2I_9$				MoS_2		
Point	$\Delta\mu_{Cs}$	$\Delta\mu_{Bi}$	$\Delta\mu_{I}$	State	$\Delta\mu_{Mo}$	$\Delta\mu_{S}$
A	−3.047	−0.046	−-0.870	S-poor	0	−1.449
B	−2.702	−0.019	−0.990	S-rich	−2.898	0
C	−3.414	−2.154	−0.278			
D	−3.687	−2.154	−0.187			
E	−3.743	−2.133	−0.173			

Table S4. The Fermi level E_F, valence band maximum level E_{VBM} and conduction band minimum level E_{CBM} at $Cs_3Bi_2I_9$ and MoS_2 sides of the $Cs_3Bi_2I_9/MoS_2$ heterostructures with different vacancy defects.

System	E_F	$E_{VBM}^{Cs_3Bi_2I_9}$	$E_{CBM}^{Cs_3Bi_2I_9}$	$E_{VBM}^{MoS_2}$	$E_{CBM}^{MoS_2}$
interface	−4.014	−4.662	−3.103	−5.179	−3.398
interface−V_{Cs}	−4.722	−5.369	−3.811	−5.886	−4.105
interface−V_I	−3.211	−3.858	−2.299	−4.376	−2.594
interface−V_S	−3.998	−4.645	−3.087	−5.162	−3.381

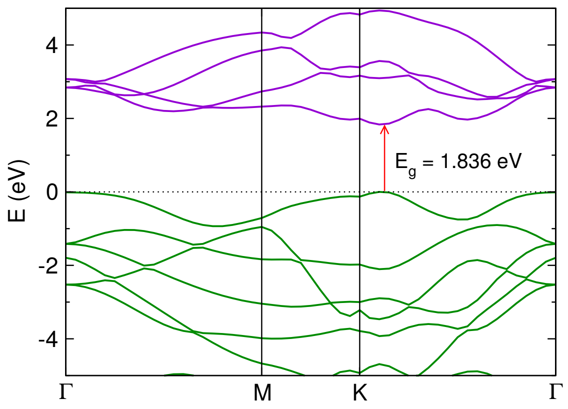

Fig. S1 Electronic band structure of MoS_2 monolayer calculated by QE package.

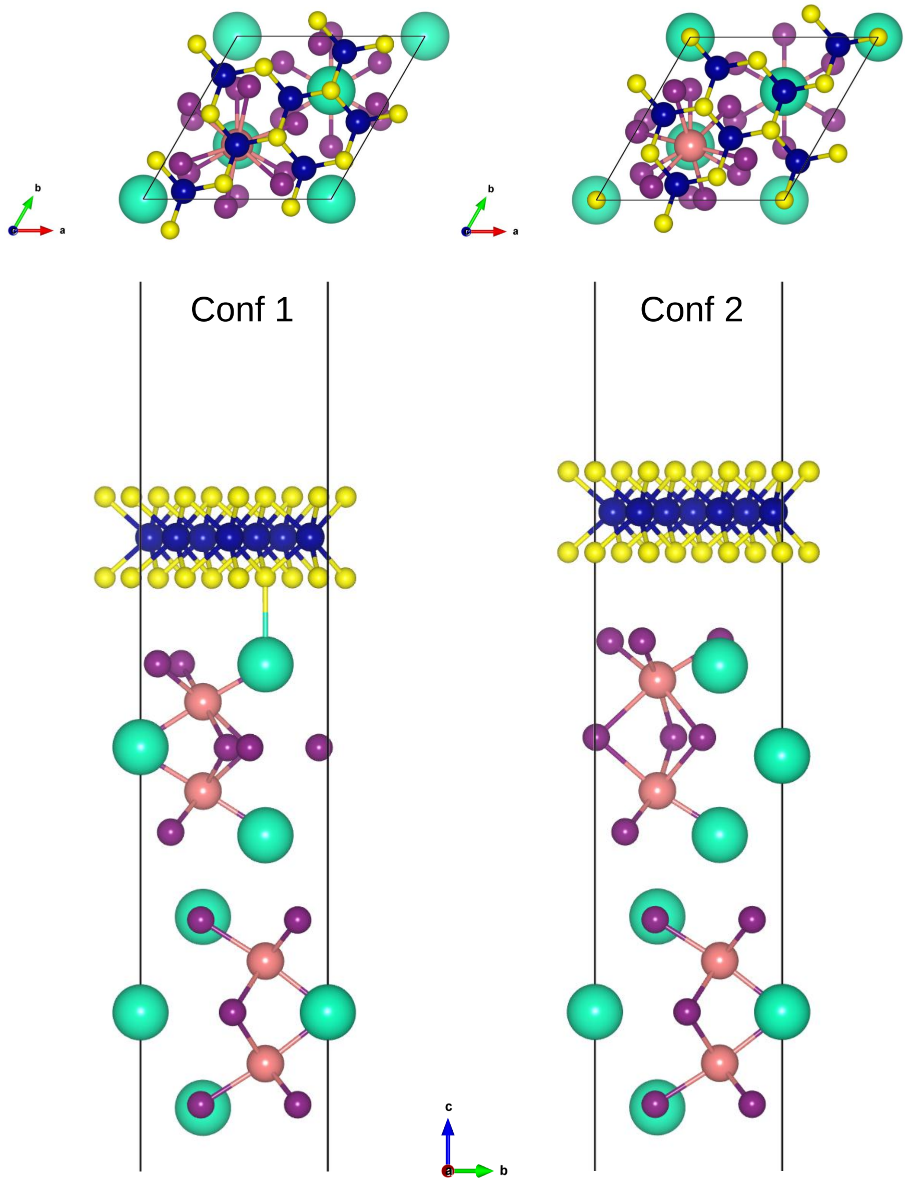

Fig. S2 Top (top panel) and side (bottom panel) views of two sliding configurations, namely Conf1 (left panel) and Conf 2 (right panel), between $Cs_3Bi_2I_9$ and MoS_2 in Cs3Bi2I9/ MoS2 heterostructure.

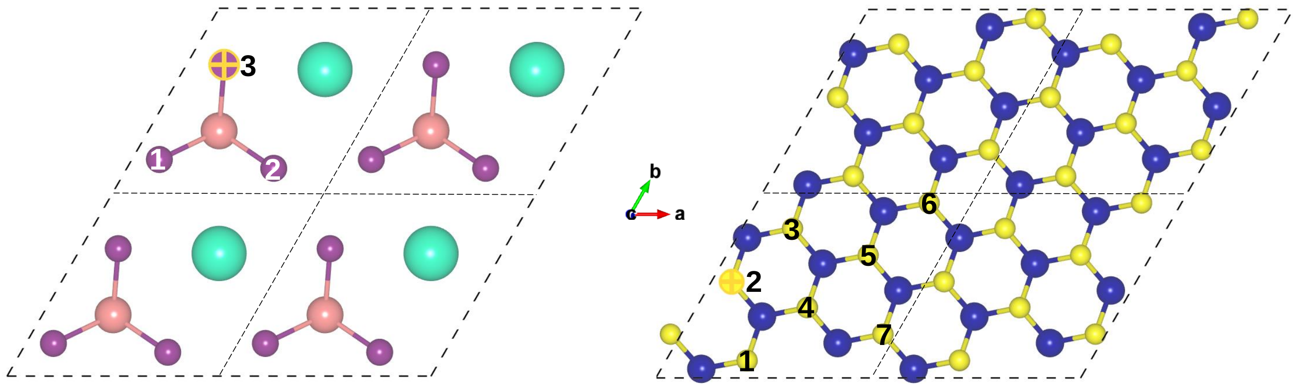

Fig. S3 All the vacancy defect sites for V_I and V_S included in the calculation of the $Cs_3Bi_2I_9/MoS_2$ heterostructure. The yellow circles with cross represent vacancy sites with the lowest energy.

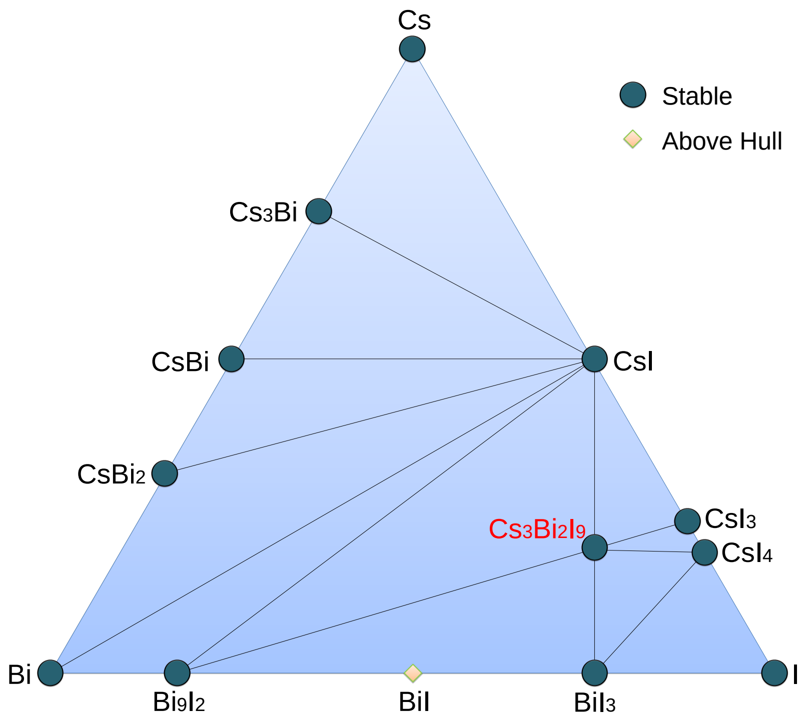

Fig. S4 A schematic of the Cs-Bi-I ternary system.

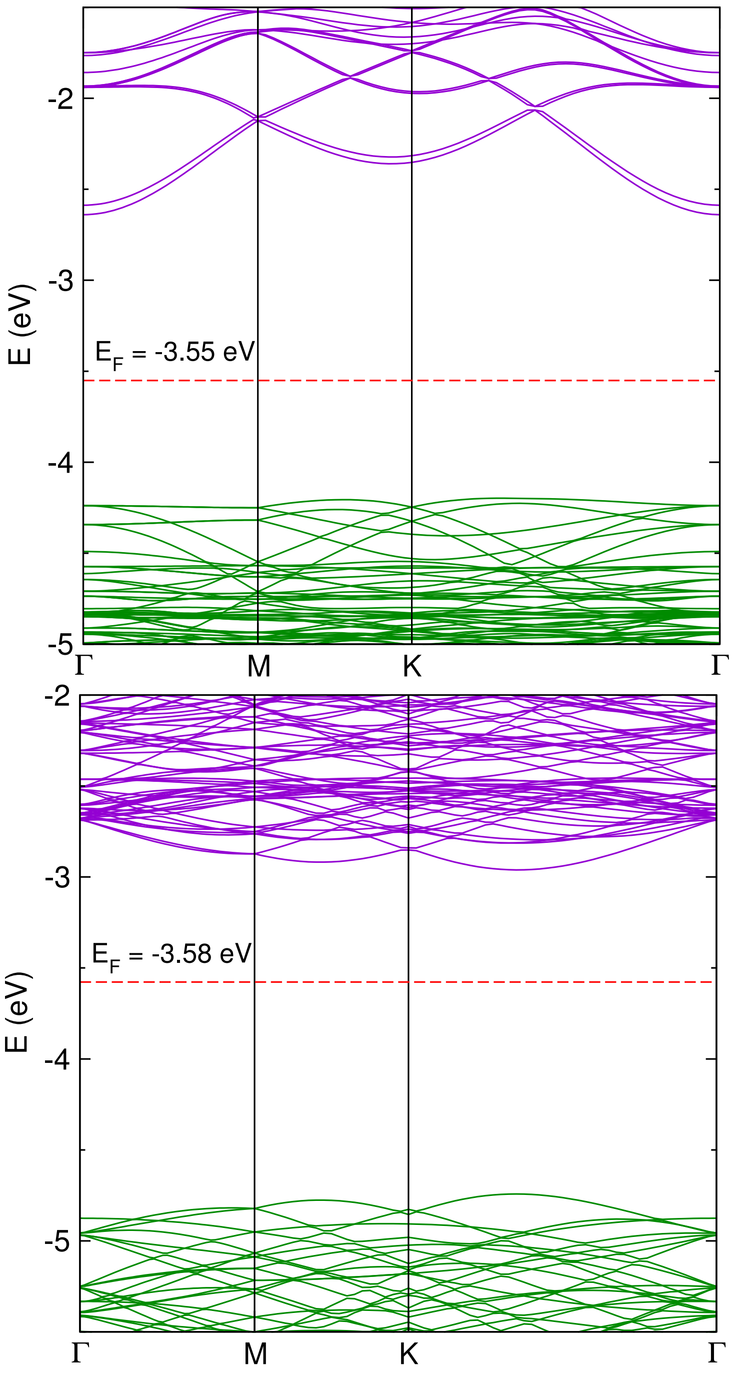

Fig. S5 Electronic band structures of $Cs_3Bi_2I_9$ and MoS_2 surfaces. The Fermi level E_F is denoted by red dashed line.

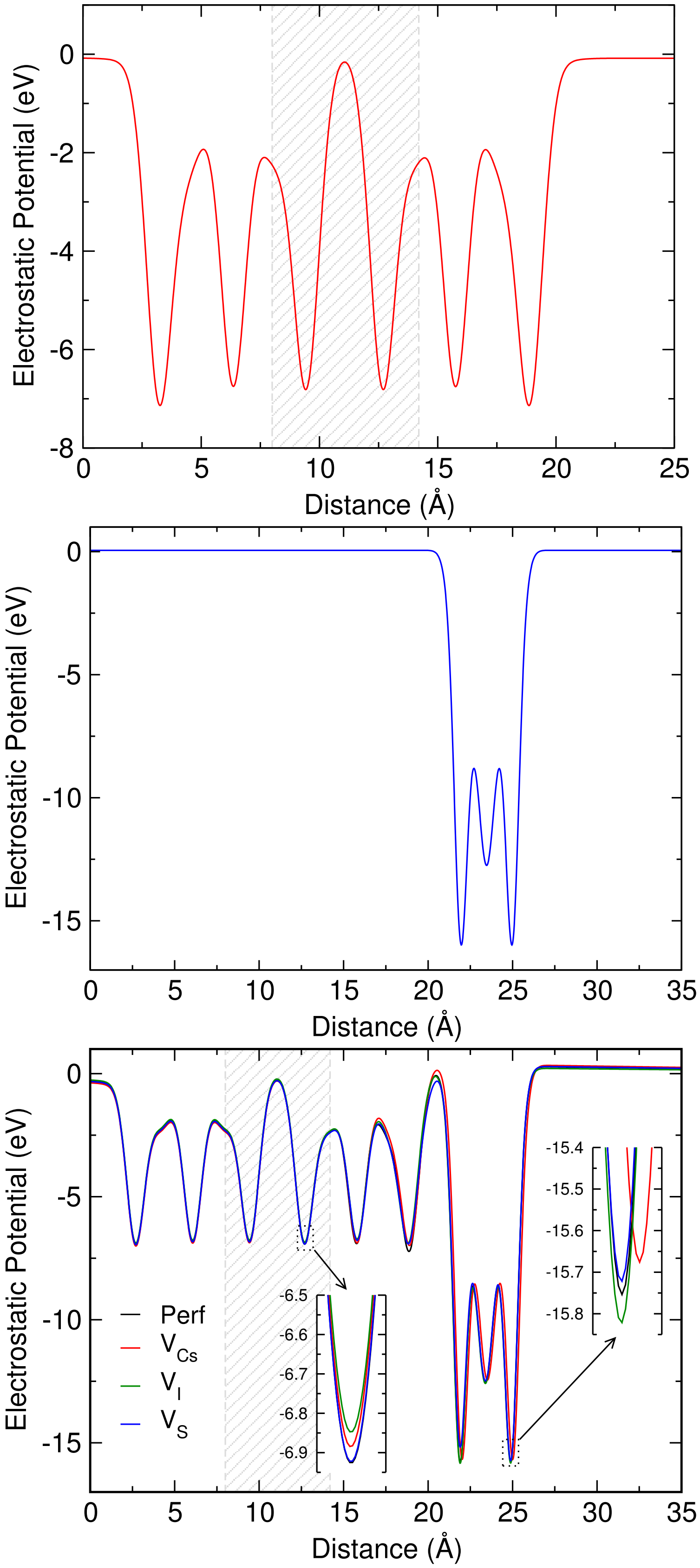

Fig. S6 Electrostatic potential in $Cs_3Bi_2I_9$ surface (top panel), MoS_2 surface (middle panel), and $Cs_3Bi_2I_9/MoS_2$ interface slab systems (bottom panel) without and with interfacial vacancy defects.

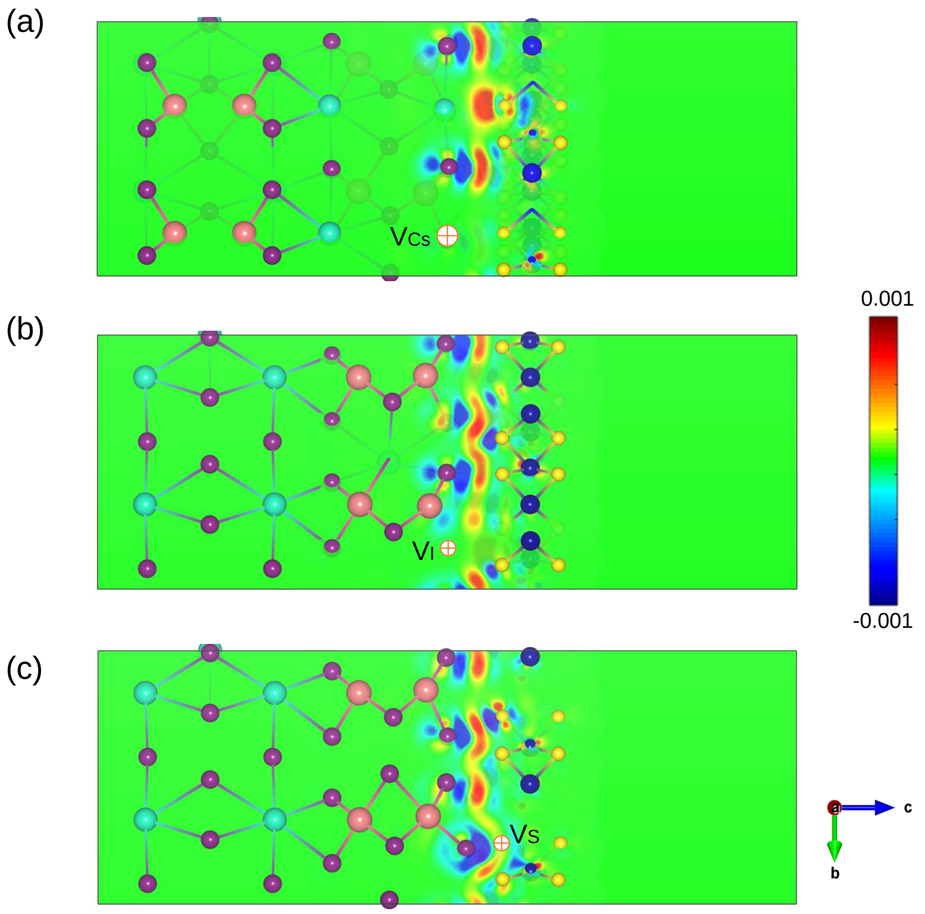

Fig. S7 Isosurface view of electron density difference at the value of 0.001|e|/$Å^3$ upon the formation of $Cs_3Bi_2I_9/MoS_2$ interfaces with V_{Cs} (a), V_I (b) and V_S (c) projected on (100) plane passing the corresponding vacancy defect site. Red (blue) colour represents the charge accumulation (depletion).

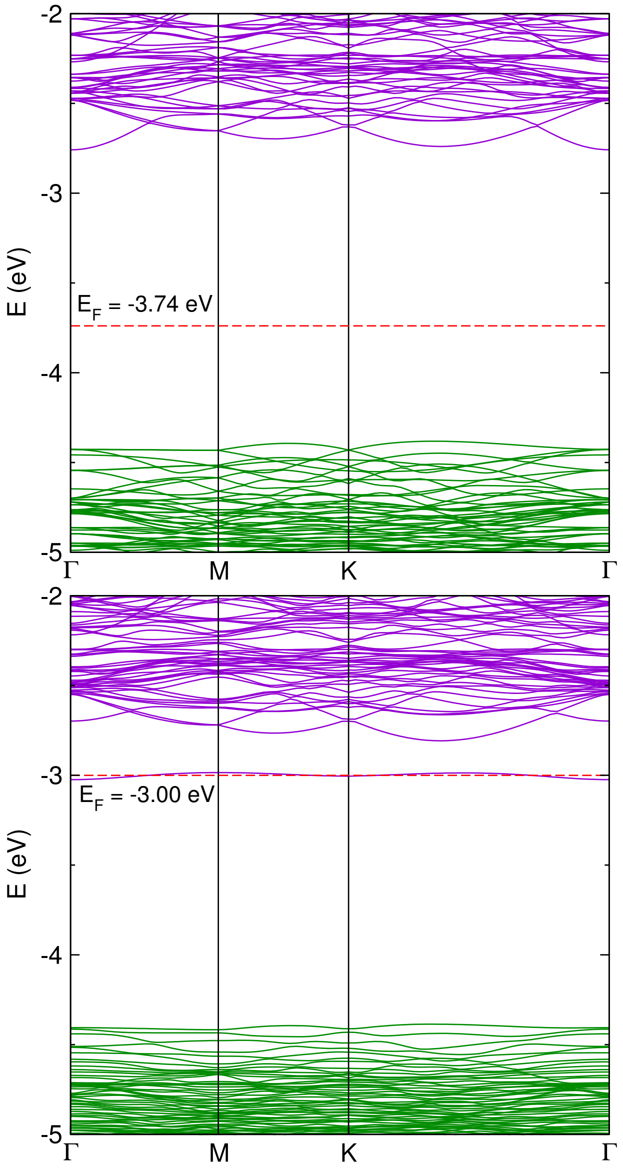

Fig. S8 Electronic band structures of $Cs_3Bi_2I_9/MoS_2$ heterostructures without any defect (top left panel), and with Cs (top right panel), I (bottom left panel) and S (bottom right panel) vacancy defect at the interface. The Fermi level E_F is denoted by red-coloured dashed line.

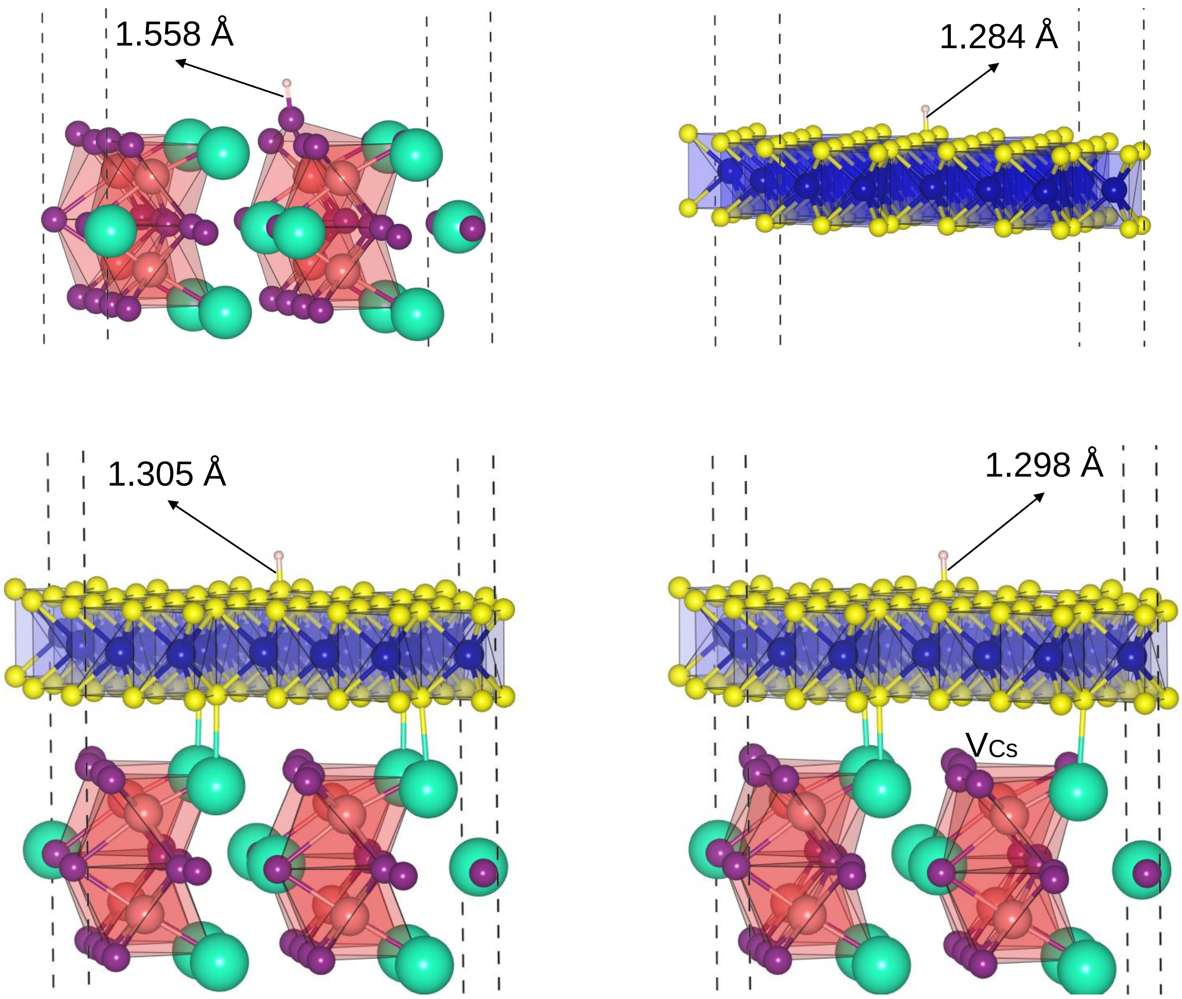

Fig. S9 Optimized geometries of the hydrogen-adsorbed $Cs_3Bi_2I_9$ surface (top left panel) and MoS_2 monolayer (top right panel), $Cs_3Bi_2I_9/MoS_2$ heterostructures without any defect (bottom left panel) and with V_{Cs} defect (bottom right panel).

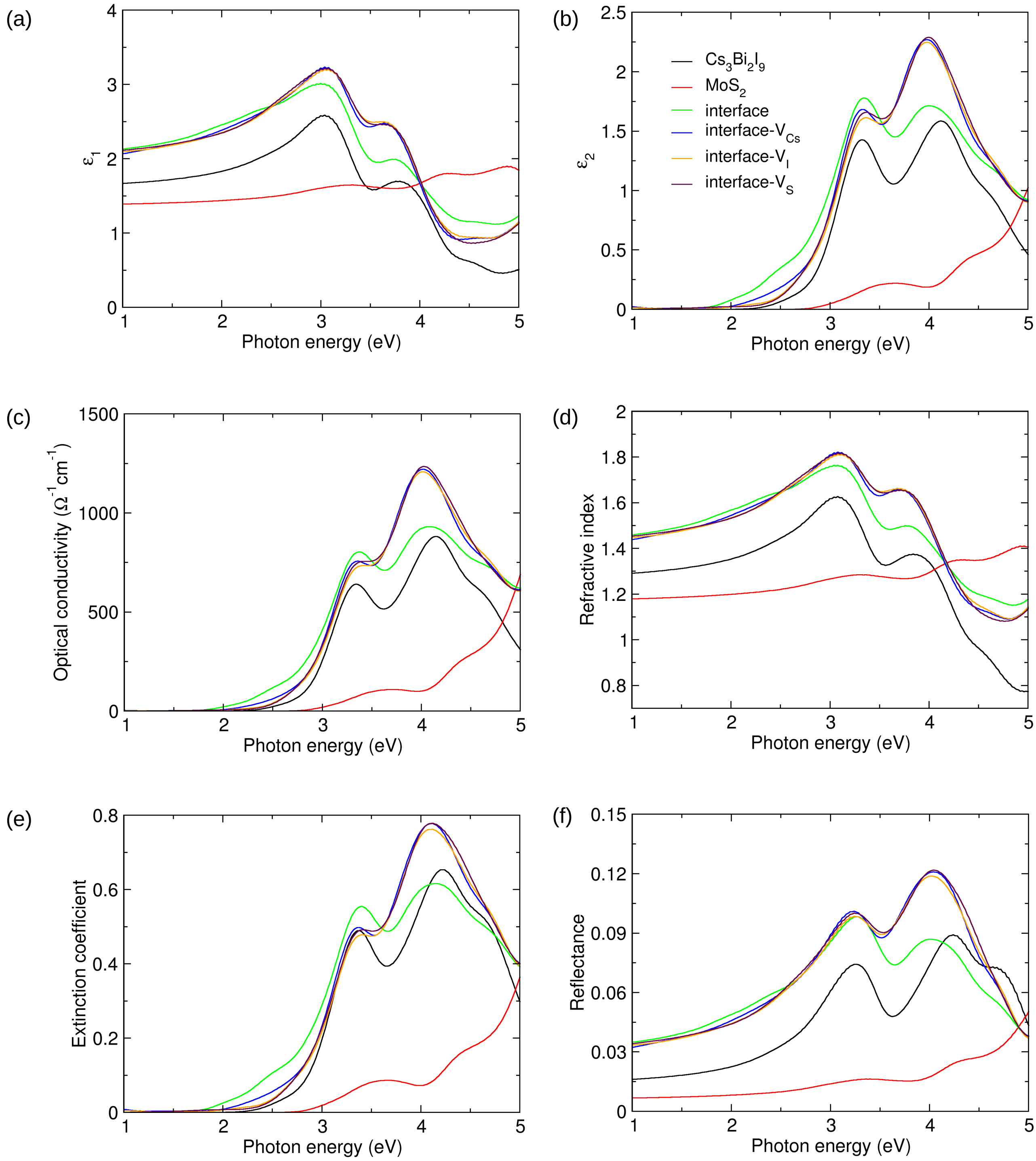

Fig. S10 Real (a) and imaginary (b) parts of the frequency-dependent dielectric functions, optical properties such as optical conductivity (c), refractive index (d), extinction coefficient (e) and reflectance (f) for $Cs_3Bi_2I_9$ and MoS_2 surface, together with their heterostructures without and with vacancy defects.

References

[1] P. Cortona, Direct determination of self-consistent total energies and charge densities of solids: a study of the cohesive properties of the alkali halides, *Phys. Rev. B3* **1992**, *46*, 2008–2014.

[2] J. Runsink, S. Swen-Walstra, T. Migchelsen, Refinement of the crystal structures of $(C_6H_5)_4AsI_3$ and CsI_3 at 20 °C and at −160 °C, *Acta Crystal. B* **1972**, *28*, 1331–1335.

[3] E. E. Havinga, Contribution to the knowledge of the structure of polyhalides, *Acta Crystal.* **1954**, *7*, 487–490.

[4] F. Emmerling, N. Laengin, D. Petri, M. Kroeker, C. Roehr, Alkalimetallbismutide ABi und ABi_2 (A = K, Rb, Cs) - Synthesen, Kristallstrukturen, Eigenschaften, *Zeit. Anorg. Allg. Chem.* **2004**, *630*, 171–178.

[5] G. Oertel, Ueber die Struktur von Caesium-Wismut-Fotokathoden, *Annal. Phys.* **1961**, *7(8)*, 137–143.

[6] G. Gnutzmann, F. W. Dorn, W. Klemm, Ueber einige A_3B- und AB_2-Verbindungen der schweren Alkalimetalle mit Elementen der V. Gruppe, *Zeit. Anorg. Allg. Chem.* **1961**, *309*, 210–225.

[7] H. G. von Schnering, H. von Benda, C. Kalveram, Wismutmonojodid Bi J, eine Verbindung mit Bi(0) und Bi(II), *Zeit. Anorg. Allg. Chem.* **1978**, *438*, 37–52.

[8] Z. G. Pinsker, The electron diffraction analysis of BiI_3 and the modern ideas on the structure of the layered lattices, *Trudy Instituta Kristallografii, Akademiya Nauk SSSR* **1952**, *7*, 35–48.

[9] E. V. Dikarev, B. A. Popovkin, Crystal structure of Bi_9I_2, *Doklady Akademii Nauk SSSR* **1990**, *310(1)*, 117–120.

[10] A. V. Arakcheeva, M. Bonin, G. Chapuis, A. I. Zaitsev, The phases of $Cs_3Bi_2I_9$ between RT and 190 K, *Zeit. Kristal.* **1999**, *214*, 279–283.

[11] K. D. Bronsema, J. L. de Boer, F. Jellinek, On the structure of molybdenum diselenide and disulfide, *Zeit. Anorg. Allg. Chem.* **1986**, *540*, 15–17.
